# Total Arsenic, Cadmium, and Lead Determination in Brazilian Rice Samples Using ICP-MS

**DOI:** 10.1155/2016/3968786

**Published:** 2016-09-28

**Authors:** Lidiane Raquel Verola Mataveli, Márcia Liane Buzzo, Luciana Juncioni de Arauz, Maria de Fátima Henriques Carvalho, Edna Emy Kumagai Arakaki, Richard Matsuzaki, Paulo Tiglea

**Affiliations:** Inorganic Contaminants Laboratory, Contaminants Center, Adolfo Lutz Institute, 355 Dr. Arnaldo Av., 01246-902 São Paulo, SP, Brazil

## Abstract

This study is aimed at investigating a suitable method for rice sample preparation as well as validating and applying the method for monitoring the concentration of total arsenic, cadmium, and lead in rice by using Inductively Coupled Plasma Mass Spectrometry (ICP-MS). Various rice sample preparation procedures were evaluated. The analytical method was validated by measuring several parameters including limit of detection (LOD), limit of quantification (LOQ), linearity, relative bias, and repeatability. Regarding the sample preparation, recoveries of spiked samples were within the acceptable range from 89.3 to 98.2% for muffle furnace, 94.2 to 103.3% for heating block, 81.0 to 115.0% for hot plate, and 92.8 to 108.2% for microwave. Validation parameters showed that the method fits for its purpose, being the total arsenic, cadmium, and lead within the Brazilian Legislation limits. The method was applied for analyzing 37 rice samples (including polished, brown, and parboiled), consumed by the Brazilian population. The total arsenic, cadmium, and lead contents were lower than the established legislative values, except for total arsenic in one brown rice sample. This study indicated the need to establish monitoring programs for emphasizing the study on this type of cereal, aiming at promoting the Public Health.

## 1. Introduction

Due to the occurrence of the industrialization and urbanization without environmental care, toxic elements such as lead (Pb), cadmium (Cd), and arsenic (As), coming mainly from mining, industrial processes, pesticides, chemical fertilizers, and atmospheric deposition, have become a major source of environmental contamination [1].

Toxic elements are considered highly hazardous to human health and they may cause acute or chronic poisoning. Chronic exposure to lead has been associated with the induction of pathological changes and damage in organs and central nervous system, leading to lower intelligence quotient in children. Cadmium is highly toxic to the kidneys and this metallic element is considered as carcinogenic. Besides, cadmium may cause bone mineralization, osteoporosis being a critical effect resulting from this element exposure. Arsenic is also considered as carcinogenic, and the majority of its chronic exposure reports are focused on skin problems like pigmentation and keratosis [1, 2].

Because of the high soil mobility and availability of the total arsenic, cadmium, and lead derived from the human activities and natural sources, there is a general concern about their phytotoxicity and risks to organisms, as they are rapidly able to spread out at different levels in the food chain [3].

It has been shown that plants growing in soils contaminated with toxic elements are not capable of preventing their uptake and accumulation in the plant tissue, but are capable of restricting them only [4]. Thus, the foods contaminated with metals have turned out to be serious problem due to the potential bioaccumulation in biosystems through contaminated water and soil. This circumstance is associated with the fact that some toxic elements are slowly eliminated from the human body, and they tend to accumulate in different tissues such as liver, muscles, and bones, threatening the human health [4].

Rice (*Oryza sativa* L.) is one of the most consumed cereals in the world [5], and it is part of the staple diet of the world population; and it is considered as the most important source of nutrients for billions of people around the world [6]. Rice provides 20.0% of energy and 15.0% of the daily requirement of protein for adults [5].

Brazil is the largest non-Asian rice producer in the world, and the average consumption of this cereal per person is nearly 25.0 kg·year^−1^ [1, 5]. According to the Brazilian Ministry of Agriculture-Livestock and Food Supply [7], Brazil imported 372,567 tons of rice and exported 961,473 tons of the cereal in 2015.

High concentrations of toxic elements are found in rice when compared to other plants grown under the normal conditions. Many toxic elements' accumulation in rice is associated with the plant characteristics and its cultivation, as it is usually grown in flooded or very humid areas, which optimize the transfer of such elements from the soil to the plant [8]. Only fish and seafood may carry higher concentrations of arsenic than rice; however, while arsenic in rice occurs mainly as inorganic arsenic species, which are very toxic, arsenic in fish and seafood occurs primarily as organic species, which are less toxic [8, 9].

In this context, the scope of this study was to investigate a suitable method for rice sample preparation, as well as to validate a method for monitoring the concentration of total arsenic, cadmium, and lead, in rice using Inductively Coupled Plasma Mass Spectrometry (ICP-MS). The proposed method was applied for analyzing different rice types (polished, brown, and parboiled) coming from various Brazilian regions in order to investigate whether they are in accordance with the national legislation, aiming at Public Health promotion.

## 2. Material and Methods

### 2.1. Reagents and Analytical Solutions

High purity deionized water (resistivity 18.2 MΩ cm^−1^) from a Millipore water purification system (Bedford, NY, USA) was used throughout this study. Lead (Pb), cadmium (Cd), arsenic (As), germanium (Ge), indium (In), and rhenium (Re) at 1000 *μ*g mL^−1^ standard solutions, produced according to the ISO Guide 34, were acquired from Inorganic Ventures (Christiansburg, VA, USA). All of the employed reagents were of analytical grade. Suprapur® HNO_3_ (65.0%) and H_2_O_2_ (30.0%) used in the digestion procedures were acquired from Merck (Darmstadt, Hesse, Germany). Instrumental daily performance solution was purchased from PerkinElmer (Shelton, CT, USA). Certified reference material IRMM 804 Rice Flour was acquired from the Joint Research Centre Institute for Reference Materials and Measurements (Geel, Antwerp, Belgium).

External calibration was performed using a five-point analytical curve, prepared by diluting the individual arsenic, cadmium, and lead standards with 5.0% (v/v) HNO_3_. Analytical curve concentrations ranged from 1.0 to 40, 1.2 to 24, and 1.0 to 20 *μ*g L^−1^ for arsenic, cadmium, and lead, respectively.

A multielement (germanium for arsenic, indium for cadmium, and rhenium for lead) internal standard solution with 5 *μ*g L^−1^ of each element in 0.2% (v/v) HNO_3_ solution was prepared by serial dilution of 1000 *μ*g mL^−1^ monoelement stock solutions. Internal standard solution was added in-line to the analyzed solutions through a mixing tee, used to blend in the internal standards with the samples after the peristaltic pumping and before the nebulizer.

### 2.2. Instrumentation

All of the measurements were conducted using an ICP-MS (Elan DRC II, PerkinElmer) instrument, equipped with a glass Meinhard® (Golden, CO, USA) nebulizer and a cyclonic glass spray chamber. A standard 2.0 mm ID quartz injector and Pt sampler (1.10 mm orifice diameter) and skimmer (0.9 mm orifice diameter) cones were used. Standard, blank, and sample solutions were delivered using a S10 (PerkinElmer) autosampler. Relevant ICP-MS operating conditions are shown in Table 1.

Quantification mode was used for determining the concentrations of elements in the solutions. Instrument performance was checked daily using a multielement standard solution of 1 *μ*g L^−1^ of Mg, In, U, Ce, and Ba (PerkinElmer). According to the instrument manufacturer, the following parameters were evaluated during the daily performance analysis: sensitivity (Mg, In, and U), doubly charges (Ba), and oxides (Ce) formation.

### 2.3. Samples and Procedures

#### 2.3.1. Rice Sample Preparation Procedures

Sample preparation procedure is a critical point for the success of the analysis [4], being considered an important source of error in analytical method development. In the present study different sample preparation procedures were investigated in order to check their suitability for rice decomposition aiming at determining the total arsenic, cadmium, and lead concentrations by means of ICP-MS.

A rice package (1 kg) of a given brand randomly selected was acquired in a market located in São Paulo city, SP, Brazil. At first, the samples were weighed using an analytical balance (Shimadzu, Kyoto, Japan) and then grinded in an IKA® analytical mill (Staufen, Baden-Württemberg, Germany), except when performing the procedure carried out in the muffle furnace, where the entire grain was ashed.

For investigating the analytes recovery for the various sample preparation procedures, the samples were spiked using arsenic, cadmium, and lead aqueous solutions. For each sample preparation procedure, three independent replicates of the sample were analyzed, and the respective procedural blanks were considered in the final results. Samples were fortified in order to get final concentrations ranging from 8.0 to 30.0 *μ*g L^−1^ (within the calibration curves concentration range) for all the analyzed elements in the final sample solutions.

#### 2.3.2. Ashing Samples in Muffle Furnace

Laboratory standard procedure for food samples mineralization was performed using a Fornitec (São Paulo, SP, Brazil) muffle furnace. In a porcelain capsule, 2.0 g of the sample was weighted and 2 mL of 60.0% Mg(NO_3_)_2_ was added. The samples were initially ignited in a Bunsen burner, and then they were taken into a muffle furnace with a heating ramp of 150°C until reaching 420°C, maintaining this temperature for 4 h. After cooling, 1 mL of HNO_3_ was added and allowed to dryness on a heating plate. Samples returned to the muffle furnace at 420°C until reaching the complete destruction of the organic matter.

Samples were spiked in order to obtain a final concentration of 9.6 *μ*g L^−1^ for arsenic, cadmium, and lead in 50 mL of at 5% (v/v) HNO_3_.

#### 2.3.3. Acid Digestion Using Metallic Block

For the metallic block [10], 1 g of the sample was weighted in a polytetrafluorethylene (PTFE) flask. HNO_3_ (1 mL) and H_2_O_2_ (2 mL) were used as reagents, and an overnight predigestion step was performed. Digestion was performed in a Marconi (Piracicaba, SP, Brazil) metallic block at 100°C for 5 hours. Samples were spiked in order to get a final concentration of 16.0, 9.6, and 8.0 *μ*g L^−1^ of arsenic, cadmium, and lead, respectively, in 25 mL of deionized water.

#### 2.3.4. Acid Digestion on Hot Plate

For digesting on the hot plate [11], 0.5 g of the sample was weighted in an Erlenmeyer flask and 1 mL of HNO_3_ and 2 mL of H_2_O_2_ were added. The mixture was left overnight and then heated on a hot plate (Marconi) at 130°C for 2 h. Samples were spiked in order to get a final concentration of 8.0 *μ*g L^−1^ of the elements in the sample solution (volume completed to 25 mL by adding deionized water).

#### 2.3.5. Microwave Digestion

For the digestion assisted by microwave radiation, the method published by Batista and coworkers [6] was performed with some modifications. In the present study, 0.5 g of rice sample was weighted and transferred to the PTFE flask specific for the microwave oven used (ETHOS ONE from Milestone, Sorisole, Bergamo, Italy). Two mL of HNO_3_, 2 mL of H_2_O_2_, and 4 mL of H_2_O were added to the sample in the flask after an overnight predigestion step. The following program was run: 1000 W at 100°C (5 min. ramp, 5 min. holding) and 1000 W at 130°C (3 min. ramp, 3 min. holding). Before digestion, samples were spiked in order to reach a final concentration of 30.0, 10.0, and 10.0 *μ*g L^−1^ of arsenic, cadmium, and lead, respectively, in the final sample solution (volume completed to 25 mL by adding deionized water).

### 2.4. Method Validation

For the method validation, the sample solution obtained from digestion in microwave oven was selected due to the lesser time consumption involved and the lower blank values. The analytical method validation was performed by considering the limit of detection (LOD), limit of quantification (LOQ), linearity, precision (repeatability), and relative bias. For all of the calculations, Eurachem [12] requirements were considered.

LODs and LOQs were established as three and ten times, respectively, the standard deviation of six rice samples independently digested considered as having approximately 1 *μ*g L^−1^ of arsenic and concentration of cadmium and lead <LOQ in the final sample solution. The final values of both parameters were calculated taking into account the samples dilution factor and the weight.

Linearity was established by preparing the calibration curves of all of the target elements by employing an unweighted least-squares linear regression.

Relative bias and repeatability of the method were evaluated by analyzing seven replicates of the certified reference material IRMM 804 Rice Flour, for determining arsenic, cadmium, and lead. Relative bias was obtained as the percentage difference between obtained and certified values, while the repeatability was calculated as relative standard deviation (RSD%).

### 2.5. Determination of Total Arsenic, Cadmium, and Lead in Rice Samples Consumed by the Brazilian Population

To evaluate the analytical method performance for determining the arsenic, cadmium, and lead in different rice types samples consumed by the Brazilian population, 37 rice samples from different brands and lots were collected from various states of the country (Ceará, Espírito Santo, Goiás, Paraíba, Rio Grande do Sul, Rondônia, and São Paulo). Samples were collected by the Health Surveillance System from 2013 to 2015 and sent to the Inorganic Contaminants Laboratory-Adolfo Lutz Institute. They consisted of 27 polished rice samples, two parboiled rice samples, and eight brown rice samples. All of the samples were collected and analyzed before their expiration dates.

### 2.6. Expanded Uncertainty Calculation

Expanded uncertainty estimation (*U*_*c*_) for the obtained results was calculated according to the criteria described in Eurachem/CITAC [13]. For this evaluation, the following parameters were considered: analytical curve uncertainty associated with the linear least-square fitting procedure; volumetric flasks calibration and temperature effect on the volumetric measurement; and analytical balance calibration.

Expanded uncertainty was determined by multiplying the coverage factor (*k*) and the combined uncertainty (*u*_*c*_): *U*_*c*_ = *k* · *u*_*c*_, considering the coverage factor *k* = 2, for a confidence level of 95.45%.

## 3. Results and Discussion

### 3.1. Sample Preparation Procedures

The well-known polyatomic interference caused by ^40^Ar^35^Cl^+^ was considered in the total arsenic determination by using arithmetic correction [14]. According to* t*-test (*p* = 0.005, *n* = 4), no statistically significant differences between corrected and uncorrected concentrations were found. Similar result was found by Cai and coworkers [15] for citrus leaves standard reference materials digested with HNO_3_ and H_2_O_2_, reagents also used in the present study. This result can be explained by the fact that only 2% of the total chlorine contents in rice plants are present in the edible part [16].

Table 2 shows the analytes recovery in the spiked samples submitted to the different preparation procedures for determining total arsenic, cadmium, and lead in rice.

Recoveries varied from 81.0 to 115.0%, indicating that results for all elements determined in the sample submitted to different preparation procedures were within the percentages recommended by FDA [17], which comprise the range between 80.0 and 120.0%. This finding highlights the possibility of using all of the sample preparation procedures employed in the present study, including arsenic determination whose volatility is well known.

Soylak and coworkers [18] also compared various sample preparation procedures for determination of trace elements in Turkey spices. Recoveries ranged from 95.0 to 97.0% for muffle oven, 99.0 to 95.0% for hot plate, and 98.0 to 103.0% for microwave oven; and these results are comparable to those found in the present investigation. The results found in the present study are in agreement with the data reported by Wei et al. [19], where acid digestion assisted by microwave radiation was carried out, reaching recoveries of 97.0, 106.0, and 103.0% for total arsenic, cadmium, and lead, respectively.

Nowadays, researches on sample preparation procedures have advanced, leaning more and more to the microwave technology, becoming difficult to find present-day publications using other procedures. The advantages of microwave technology are undeniable, but the use of less resourceful instruments may eventually be appropriate, providing good results for the analytes under study.

### 3.2. Method Validation Studies

The overall performance of the proposed method for total arsenic, cadmium, and lead determination in rice is summarized in Tables 3 and 4.

LODs and LOQs results were considered acceptable as the values were below the limits established by the Brazilian legislation for arsenic, lead, and cadmium for rice and its derivatives: 0.30 mg kg^−1^, 0.20 mg kg^−1^, and 0.40 mg kg^−1^, respectively [20].

The linearity of the calibration curve was evaluated by investigating the correlation coefficient (*r*) of the calibration curves. The *r* values were higher than 0.998 [19] for all of the analytes, as shown in Table 3, and the regression analyses showed that the linear correlation between concentration and signal intensity was significant (*p* < 0.05).

Relative bias (Table 4) was assessed through the difference between the mean value of obtained results and the certified values. Bias was considered acceptable since it varied less than ±20.0% of the target value [19]. For all elements, the RSD (repeatability) was in compliance with the European Commission Decision 2002 [48], and it did not exceed 10.0%. Therefore, as expected, the use of microwave oven for rice acid digestion was appropriate for determining the total arsenic, cadmium, and lead concentrations in rice using ICP-MS.

### 3.3. Method Application

The above was method applied for quantifying the concentration of total arsenic, cadmium, and lead in 37 rice samples (polished, parboiled, and brown rice). Results are summarized in Table 5.

According to Table 5, of 37 analyzed samples, 35 showed total arsenic concentration higher than the LOQ, ranging from 0.061 to 0.660 mg kg^−1^.

In two polished rice samples, total arsenic concentration was lower than LOQ. The average total arsenic concentration in parboiled rice was lower than that found in other rice types (0.071 mg kg^−1^), followed by polished rice (0.130 mg kg^−1^) and brown rice, which had the highest arsenic concentration (0.224 mg kg^−1^).

In one brown rice sample, collected in the state of São Paulo, the total arsenic concentration was 0.660 mg kg^−1^. This value was higher than that established by the Brazilian legislation [20] and the Codex Alimentarius [49], which is 0.30 mg kg^−1^. This sample was considered unsatisfactory for human consumption. Excepting the brown rice sample, total arsenic concentrations were in agreement with the maximum level established for total arsenic in raw rice.

Total arsenic mean concentrations found in the present study were in agreement with those cited in the literature; and the respective authors pointed out the evident occurrence of the highest arsenic concentrations in brown rice. This occurs owing to the fact that rice plants concentrate this element in the outer layer of the grain, in the region corresponding to the pericarp. A great amount of arsenic is removed during the rice polishing [21, 50–52]. Besides, variation in the arsenic concentration may occur due to different cultivars, geography, environment, water quality, and growth conditions [21, 24, 25, 29, 30, 37, 51, 53–56].

The results obtained in the present work are comparable to others in investigations performed in Brazil, as shown in Table 6. Usually, the data from several studies presented the same tendency, the total arsenic concentration being higher in brown rice, followed by polished and parboiled rice.

The results found in this study on the total arsenic contents were also in agreement with those previously reported for rice analyzed in other countries (Table 7). The exception is for arsenic concentration found in rice cultivated in Bangladesh, one of the countries that have been mostly vulnerable to arsenic exposure, causing a serious Public Health problem [33, 34, 53]. In the 1970s, the plumbing pipes contaminated by arsenic were installed in the country, and it caused the contamination of the water consumed by the population. In consequence, it originated the contamination water used for rice irrigation.

With respect to cadmium, only one polished rice sample had cadmium concentration above the LOQ (0.042 ± 0.008 mg kg^−1^). In the other rice samples, cadmium concentration was below the LOD in 17 samples, while in 19 samples it was below the LOQ. In none of the analyzed samples, cadmium concentrations exceeded the maximum value of 0.40 mg kg^−1^ allowed by the Brazilian legislation [20] and the Codex Alimentarius [57]. Thus, referring to cadmium contamination, the polished, parboiled, and brown rice samples were considered as satisfactory for consumption.

Cadmium concentration found in the analyzed samples were similar to those reported in other studies carried out by Brazilian investigators, as outlined in Table 6. In all of the investigations, the cadmium levels were lower than those recommended by the Brazilian legislation. The same is observed when the data on rice from other countries are compared (Table 7). However, higher cadmium concentrations in rice have been reported, which can be attributed to the soil contaminated by metals in mining and industrial areas [31, 56, 58, 59].

As for lead, in 14 polished rice and one parboiled rice samples lead concentration was below the LOD, and 11 polished rice and four brown rice samples had lead concentrations below the LOQ. In one parboiled rice (0.127 mg kg^−1^), two polished rice (0.087–0.115 mg kg^−1^), and four brown rice (0.065–0.124 mg kg^−1^) samples, the lead concentrations were above LOQ, with mean values of 0.101 mg kg^−1^ for polished rice and 0.104 mg kg^−1^ for brown rice.

In all of the analyzed samples, the results indicated that the lead concentrations were lower than the maximum value allowed, which is 0.20 mg kg^−1^, according to the Brazilian legislation [20] and European Community [60]. Thus, regarding to lead, the Brazilian rice evaluated in this study was shown to be suitable for consumption. The lead concentrations found in Brazilian rice (Table 6) and other countries (Table 7) were comparable, not exceeding the maximum value established by the Brazilian legislation [20]. Higher lead contents found in some investigations might indicate the soil contamination by fertilizers, industrial, and/or mining activities [31, 56, 59].

## 4. Conclusions

Regarding the sample preparation, all of the procedures (ashing in muffle furnace, acid digestion under heating on metallic block or hot plate, and acid digestion assisted by microwave radiation) led to good results (recoveries ranging from 80.0 to 120.0%). This information is important for laboratories that execute rice analyses, as the sample preparation may be cheaper. The parameters obtained for validation have demonstrated that the method fits for its purpose, which is to quantify the total arsenic, cadmium, and lead in complying with the Brazilian legislation limits. Regarding the parboiled, polished, and brown rice samples analyzed, the concentrations of total arsenic, cadmium, and lead were lower than the established limits according to the Brazilian legislation, Codex Alimentarius, and European Community, except for total arsenic in one sample. The other analyzed rice samples were considered satisfactory for human consumption regarding the investigated elements.

The data obtained indicated that only the consumption of brown rice can represent risk to Brazilian population. Therefore, it is necessary to establish programs to focus attention on this type of rice aiming at promoting the Public Health, since rice might be an important route for human exposure to toxic elements, particularly in populations following diets based on this cereal, as is the case of the Brazilian population.

## Figures and Tables

**Table 1:** Inductively Coupled Plasma Mass Spectrometry (ICP-MS) operating conditions.

RF power, W	1400
Plasma gas flow rate, L min^−1^	18
Auxiliary gas flow rate, L min^−1^	1
Nebulizer gas flow rate, L min^−1^	1.02
Integration time, ms	750 (per isotope)
Isotopes monitored	^72^Ge^*∗*^, ^75^As, ^111^Cd, ^115^In^*∗*^, ^187^Re^*∗*^, ^206^Pb^*∗∗*^, ^207^Pb^*∗∗*^, ^208^Pb^*∗∗*^

^*∗*^Internal standards.

^*∗∗*^When referring to Pb concentration, the values are expressed as the mean between the three isotopes.

**Table 2:** Recovery of total arsenic, cadmium, and lead from rice sample submitted to different preparation procedures (ashing in muffle oven, acid digestion under heating on metallic block or hot plate, and acid digestion assisted by microwave radiation). The analytes concentrations were determined using ICP-MS.

Preparation procedure	Recovery (%)		
	Arsenic	Cadmium	Lead
Muffle oven	91.7	89.3	98.2
Metallic block	103.3	94.2	95.7
Hot plate	115.0	81.0	102.0
Microwave	108.7	110.2	92.8

**Table 3:** LOD, LOQ, working range, and correlation coefficient of calibration curve for the microwave digestion method.

Parameters (unit)	As	Cd	Pb
LOD (mg kg^−1^)^*∗*^	0.011	0.012	0.030
LOQ (mg kg^−1^)^*∗*^	0.038	0.040	0.064
Working range (*µ*g L^−1^)	1–40	1.2–24	1–20
Correlation coefficient of the calibration curve	0.9997	0.9997	0.9991, 0.9991, 0.9992

^*∗*^For Pb, calculated as the mean values among the three measured isotopes cited in Table 1.

**Table 4:** Certified value, obtained value, relative bias (%), and repeatability (RSD%) obtained for IRMM 804 acid digested in microwave oven.

Element	Certified value (mg kg^−1^)	Uncertainty (mg kg^−1^)^*∗*^	Obtained value (mg kg^−1^)^*∗∗*^	Standard deviation (mg kg^−1^)	Bias (%)^*∗∗*^	Repeatability (RSD%)
As	0.049	0.004	0.051	0.001	4.1	1
Cd	1.61	0.07	1.60	0.05	−0.6	3
Pb	0.42	0.07	0.44	0.04	4.8	10

^*∗*^As indicated in the IRMM 804 certificate.

^*∗∗*^Calculated as the mean of seven independent replicates of the IRMM 804.

**Table 5:** Concentrations of total arsenic, cadmium, and lead in the analyzed rice samples (average values, *n* = 3 ± *U*_*c*_^*∗*^).

Rice type	As	Cd	Pb
	mg kg^−1^		
Parboiled	0.061 ± 0.008	<0.040^b^	<0.030^a^
Parboiled	0.080 ± 0.008	<0.040^b^	0.127 ± 0.027
Polished	0.074 ± 0.008	<0.040^b^	<0.030^a^
Polished	0.062 ± 0.008	<0.040^b^	<0.030^a^
Polished	0.232 ± 0.007	0.042 ± 0.008	<0.030^a^
Polished	0.147 ± 0.008	<0.040^b^	<0.030^a^
Polished	0.135 ± 0.008	<0.040^b^	<0.064^b^
Polished	0.128 ± 0.008	<0.012^a^	<0.030^a^
Polished	0.156 ± 0.008	<0.040^b^	<0.030^a^
Polished	0.181 ± 0.008	<0.012^a^	<0.030^a^
Polished	0.133 ± 0.008	<0.012^a^	<0.030^a^
Polished	0.172 ± 0.008	<0.012^a^	<0.030^a^
Polished	0.109 ± 0.008	<0.012^a^	<0.030^a^
Polished	0.211 ± 0.007	<0.040^b^	<0.030^a^
Polished	0.178 ± 0.008	<0.040^b^	0.087 ± 0.028
Polished	0.125 ± 0.008	<0.012^a^	<0.064^b^
Polished	0.108 ± 0.008	<0.012^a^	0.115 ± 0.027
Polished	0.062 ± 0.008	<0.040^b^	<0.064^b^
Polished	0.157 ± 0.008	<0.040^b^	<0.064^b^
Polished	0.155 ± 0.008	<0.012^a^	<0.064^b^
Polished	0.167 ± 0.008	<0.040^b^	<0.064^b^
Polished	0.116 ± 0.008	<0.012^a^	<0.064^b^
Polished	0.119 ± 0.008	<0.012^a^	<0.064^b^
Polished	0.113 ± 0.008	<0.012^a^	<0.064^b^
Polished	0.166 ± 0.008	<0.012^a^	<0.030^a^
Polished	0.131 ± 0.008	<0.012^a^	<0.064^b^
Polished	0.245 ± 0.007	<0.012^a^	<0.030^a^
Polished	<0.038^b^	<0.012^a^	<0.030^a^
Polished	<0.038^b^	<0.040^b^	<0.064^b^
Brown	0.165 ± 0.008	<0.040^b^	<0.064^b^
Brown	0.160 ± 0.008	<0.040^b^	0.104 ± 0.027
Brown	0.660 ± 0.007	<0.040^b^	<0.064^b^
Brown	0.175 ± 0.008	<0.040^b^	<0.064^b^
Brown	0.166 ± 0.008	<0.040^b^	0.065 ± 0.028
Brown	0.190 ± 0.007	<0.012^a^	0.124 ± 0.027
Brown	0.180 ± 0.008	<0.040^b^	0.123 ± 0.027
Brown	0.101 ± 0.008	<0.012^a^	<0.064^b^

^a^LOD, ^b^LOQ.

^*∗*^ *U*_*c*_: expanded uncertainty, coverage factor *k* = 2, for a confidence level of 95.45%.

**Table 6:** Total arsenic, cadmium, and lead concentrations in different types of rice consumed in Brazil, reported in the literature (mg kg^−1^).

Reference	Element	Element concentration in different rice types (mg kg^−1^)		
		Polished	Brown	Parboiled
[6]	Pb	0.0053	0.0078	0.0031

[21]	As	0.223	0.348	0.215

[22]	As	0.153	0.133	0.168

[23]	As	<0.0898	<0.0898	<0.0898
	Cd	0.0078	0.0160	0.0109
	Pb	0.149	0.260	0.120

[24]	As	0.184–0.208	—	—
	Cd	0.0114–0.0119		
	Pb	0.00218–0.00421		

[25]	As	0.164	0.293	—
	Cd	0.0189	0.0168	—
	Pb	0.057	0.109	—

[26]	Cd	0.036	0.023	—

[27]	As	0.13–0.47	0.13–0.23	0.12–0.28

[28]	Cd	<0.002	—	<0.002
	Pb	<0.04	—	<0.04

Present study	As	0.144	0.272	0.071
	Cd	<0.040	<0.040	<0.040
	Pb	0.097	0.104	0.078

**Table 7:** Concentrations of total arsenic, cadmium, and lead in rice consumed by the population of various countries, reported in the literature.

Country	Element concentration (mg kg^−1^)			Reference
	As	Cd	Pb	
China	0.080	0.037	0.060	[29]
	0.336	0.312	0.022	[30]
	—	0.15	—	[31]
	—	—	0.034–0.076	[32]

Bangladesh	0.32	—	—	[33]
	0.321	0.088	0.713	[34]

India	0.051	0.019	2.277	[35]

Iran	—	<0.015	0.320	[36]
	0.121	—	—	[37]
	0.065	—	—	[38]

United Kingdom	0.187	—	—	[39]
	0.124 (polished)	—	—	[40]
	0.205 (brown)			

Spain	0.1695	—	—	[41]

Italy	0.18–0.28	0.01–0.08	—	[42]

Thailand	0.139–0.239	—	—	[43]

Japan	0.032–0.239	0.013–0.043	—	[44]

Turkey	0.0985	0.314	—	[45]

Greece	0.042–0.271	—	—	[46]

Jamaica	0.11–0.165	<0.040–0.033	—	[47]
